# The feeding apparatus of ants: an overview of structure and function

**DOI:** 10.1098/rstb.2022.0556

**Published:** 2023-12-04

**Authors:** Adrian Richter, Evan P. Economo

**Affiliations:** Biodiversity and Biocomplexity Unit, Okinawa Institute of Science and Technology Graduate University, 1919-1, Tancha, Onna-son, Okinawa 904-0495, Japan

**Keywords:** feeding performance, food uptake, functional morphology, µCT-scan, Formicidae

## Abstract

Ants are a dominant family of eusocial terrestrial insects with a diversity of ecologies, lifestyles and morphologies. Ant diet preferences range from strict carnivory through omnivory to almost complete herbivory in species feeding on seeds or exudates of plant-sucking insects. While several studies have investigated ant feeding performance on different substrates, comparatively little is known about the functional morphology of the structures involved in food uptake or their diversification across the ants. To take stock of our current knowledge, we give an overview of how adult ants ingest food, followed by a morphological description of the mouthparts, preoral space and cephalic sucking pump. The mandibles are the most prominent mouthparts and have received considerable attention in the literature, so we focus on the maxillae and labium here. We present current hypotheses for the movement patterns of these parts and discuss morphological differences among ants that may be related to their ecological diversity. Finally, we give short comparisons of the ant condition with some other insects and vertebrates, as well as an outlook summarizing gaps in our knowledge. This sets the stage for future studies elucidating the connections between ant feeding mechanisms and mouthpart evolution.

This article is part of the theme issue ‘Food processing and nutritional assimilation in animals’.

## 1. Introduction

Ants are dominant terrestrial arthropods with a biomass exceeding that of wild mammals and birds [1]. As ‘ecosystem engineers' ants have a huge impact on the ecosystems that they live in [2], with implications for human societies [3]. With currently 14 112 valid species [4], ants are the most diverse eusocial insects, exhibiting a wide range of ecological preferences and lifestyles [5]. While most ants are omnivores and can exploit both plant- and animal-based food sources [6], many have strong food preferences or specializations. Some are pure generalized or specialized predators [7], including egg predation [8], while others feed facultatively on wild mushrooms [9], or on cultivated fungus combined with juices of plants used as fungus substrate [10]. Species that are almost purely herbivorous may feed on the excretions of plant sucking insects and extrafloral nectaries [11], or on seeds [12]. All of this raises the question: Is the wide ecological spectrum of ants reflected in the morphological structures used for food processing and uptake?

The physical basis for feeding in ants and other insects are the mouthparts, the preoral space that they enclose, and a cephalic sucking pump of the foregut [13]. The mouthparts consist of a pair of mandibles, a labrum, a pair of maxillae and a labium. Mouthpart morphology in insects can be described as ‘variations on a theme', as these general components can be modified in almost any way imaginable, from piercing stylets to cutting scissors, sucking proboscises or soft lapping tongues. A general overview can be found, e.g., in [13].

For ants, mouthpart anatomy was described in 1877 by Lubbock [14]. However, the most detailed documentation for a century came later, from French anatomist Charles Janet in his work on the head of *Lasius niger* [15]. Bugnion [16] presented a more comparative contribution, focusing specifically on the feeding-related structures for 11 species of the five most speciose ant subfamilies. He provided little information on muscles but drew the structures in their natural configuration. Gotwald [17] instead studied disarticulated mouthparts of 104 species (belonging to 11 of 16 ant subfamilies), obfuscating the close integration between structures. Only recently, a new wave of studies has provided detailed accounts of skeletomusculature of various ant species using µCT-scan-based 3D reconstructions [18–23].

Food uptake performance of ants is crucial for their ecology and evolution [24]. While parameters such as uptake rate and feeding duration on fluids have been investigated in diverse contexts, e.g., [24–29], only a single study has attempted to relate feeding apparatus morphology to ecological preferences so far [30]. Similarly, only one study addresses functional morphology of ant feeding structures other than the mandibles [31], leading to a very limited understanding of their biomechanics. The mandibles have received much more attention comparatively (e.g. [7,32–35], including biomechanical investigations [36,37].

Here, we aim to provide an overview of our current knowledge of how ants take up food, including summaries of morphology, function and variation of the various feeding structures. As the mandibles are treated in two other contributions to this issue [38,39] and have received considerable previous attention in the literature, we provide only a short section on them. To provide additional context to the ant condition, we briefly compare it with a few other insects as well as vertebrates, and finally give an outlook on some potential future research areas.

## 2. Material and methods

### (a) Literature review

We performed literature searches on Google Scholar with prompts on each individual ant mouthpart (e.g., maxilla AND Formicidae AND [anatomy OR morphology]) and scanned the first 100 articles based on titles to find studies focused on ant mouthpart morphology and/or function. When we found such articles, we scanned their literature sections and the articles that cited them for other publications mentioning ant mouthparts. As we focused our search on articles that directly deal with mouthparts or their function, our literature selection is not exhaustive. Our main references for the ant feeding process are Tschinkel & Kwapich [12] Josens *et al.* [26] and Paul & Roces [29,30] and articles citing or being cited by them. General morphological descriptions are based on Janet [15], Bugnion [16] and Richter *et al*. [21], statements on mouthpart variability mainly on Gotwald [17], Richter *et al*. [20–23,40] and mouthpart function on Paul *et al*. [31]. Additional information on variation in the preoral cavity is drawn from Febvay & Kermarrec [41], Hansen *et al*. [42] and Wang *et al*. [43,44].

### (b) Visualization

To visualize the ant feeding system, we created 3D renders based on micro-computed tomography (µCT) scans of *Leptomyrmex unicolor* Emery, 1895 (Formicidae, Dolichoderinae) and *Formica rufa* Linnaeus, 1761 (Formicidae, Formicinae), both used in previous work [21,45] and published on Zenodo. Both species are omnivores, with a significant amount of arthropod prey but also nectar and in the case of *Formica* aphid exudates as part of their diet. While workers of *F. rufa* usually form large trails and forage in groups, those of *L. unicolor* are often single foragers and scavengers, although they may also recruit nest-mates to rich food sources [46,47].

**Figure 1.**
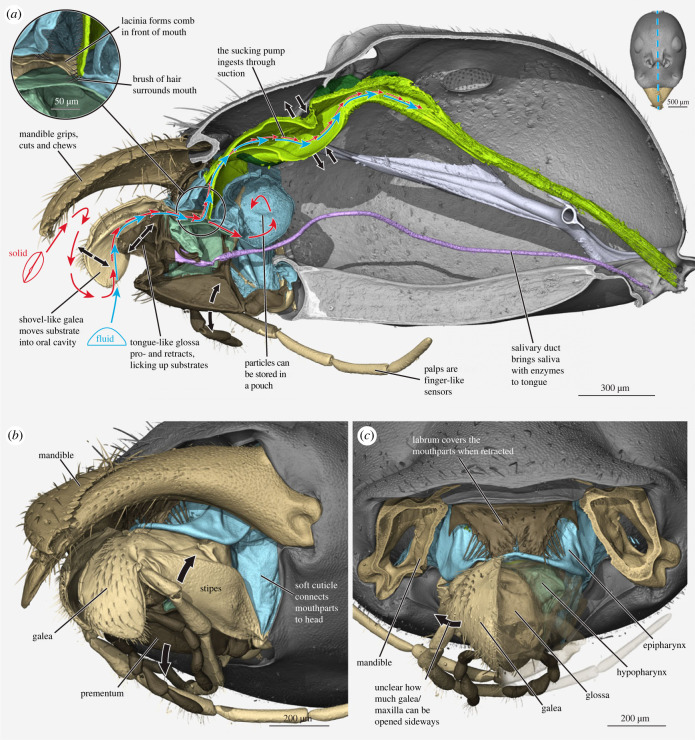
Micro-computed tomography (µCT)-based 3D reconstructions of the ant feeding apparatus (*Leptomyrmex unicolor*), illustrating our current ideas of mouthpart movements. (*a*) Sagittal section through the head (position of section marked on upper right), highlighting the most likely path of food. Red arrows for solid food, cyan for fluids. Solids are first gripped and processed by the mandibles before transport into the oral cavity by labium and maxillae. Larger particles are filtered at the mouth opening (upper left insert) and stored in the infrabuccal pouch. Hairs of mouth filter not visible in rendering, indicated by drawing. Smaller particles and possibly pre-digested substrate from the pouch pass the filter and are taken up by the sucking pump. Fluids are licked up by the glossa and directly pass the filter. Some prominent movements based mostly on Paul *et al*. [30,31] are indicated by black arrows: galea is moved up and down, glossa is extended and retracted, whole maxillolabial complex can be rotated outwards or inwards, sucking pump can be extended and compressed. (*b*) Mouthparts in oblique side view. Maxillolabial complex is partly extended, the galea overhangs the labium. (*c*) Frontal view of mouthparts with mandibles cut at the base and left maxilla transparent. Sidewards arrows indicate potential outwards movement of maxilla, but maxillolabial fusion likely restricts this direction. Colours: beige: maxillae; blue: membranes of the oral cavity; brown: labrum; dark brown: labium; green: sucking pump; grey: head capsule; purple: salivary duct; turquoise: hypopharynx.

**Figure 2.**
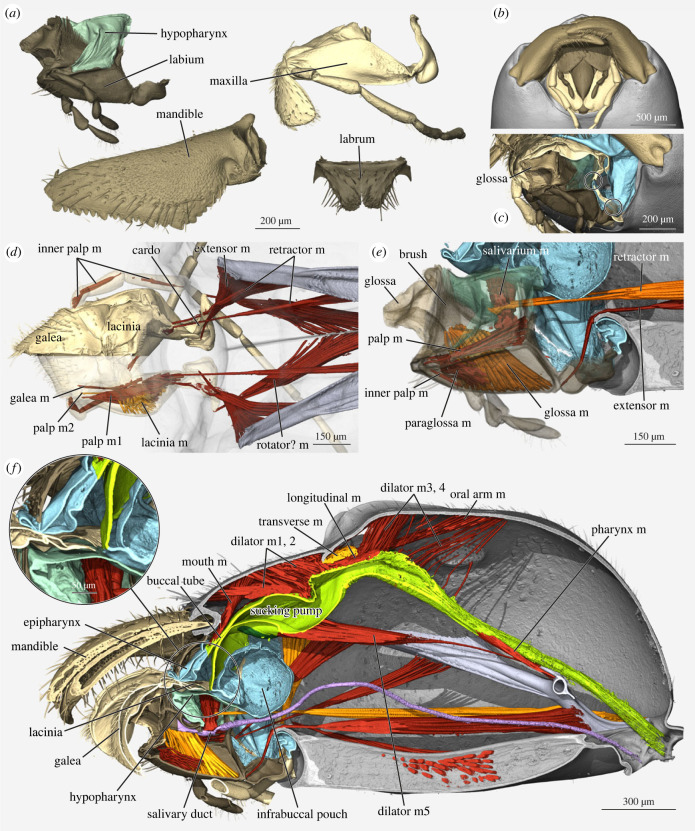
Micro-computed tomography (µCT)-based 3D reconstructions of the ant feeding apparatus anatomy. *Formica rufa* in (*c*) and *Leptomyrmex unicolor* elsewhere. (*a*) General ant mouthparts shown individually. Labium lateral, mandible dorsal, maxilla ventral, labrum frontal view. (*b*) Frontoventral view of closed mouthparts. Labrum covers part of maxillolabial complex and preoral space is sealed off. (*c*) The same view as figure 1*b*, but maxilla partly cut away to reveal points of fusion with labium (circles). (*d*) Musculature of maxillae seen from above, maxilla transparent in lower part. (*e*) Labium-associated muscles in side view, labium and hypopharynx transparent. (*f*) Head in sagittal section, focus on sucking pump and its muscles. Insert shows mouth opening with lacinial comb as filter. Colours: grey: head capsule and tentorium; light brown: mandibles; dark brown: labrum and labium; beige: maxilla; light blue: soft cuticle of preoral space; turquoise: hypopharynx; green: sucking pump; purple: salivary duct; red and orange: muscles. Interactive 3D models to further explore mouthpart anatomy are available here: mouthparts**:** https://skfb.ly/oHJoZ; maxilla: https://skfb.ly/oHJpp; labium: https://skfb.ly/oHJp7; sucking pump: https://skfb.ly/oHJpr.

**Figure 3.**
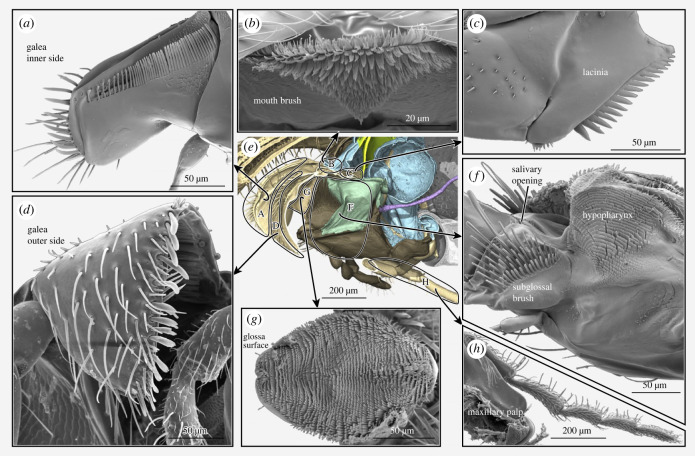
Impressions of cuticular hairs on mouthparts and preoral cavity of ants based on electron microscopy images. (*a–c,f,g*) *Brachyponera luteipes*, (*d*) *Dolichoderus laminatus*, (*e*) *Leptomyrmex unicolor*, (*h*) *Formica rufa*. (*e*) Overview of the mouthparts based on a 3D rendering (figure 1), positions of structures are circled, curves of connecting arrows indicate viewing direction. (*a*) Inner galea side with dense comb of hairs. (*b*) Brush of hairs in front of mouth. (*c*) Lacinia with dense comb of spines along margin. (*d*) Outer side of galea covered with hairs, different hair types on medial margin. (*f*) Side of the labium, showing the hypopharynx covered in tiny hairs, the thick brush behind the glossa and the salivary opening. (*g*) Glossa surface. (*h*) Maxillary palps covered in sensory hairs. Anterior always facing left, except (*b*) and (*d*)*,* which are in anterior view, lateral to the left in (*d*). (*d*) Taken from Keller [40], accessed through www.Antweb.org, specimen number ANTWEB1008520.

The µCT scan data were processed in Amira 2020.2 (Visage Imaging, Berlin, Germany), segmenting individual structures into materials. Structures were first marked on every 20th slice and the segmentation was then semi-automatically completed using Biomedisa [48]. Finally, the resulting segmentation was manually cleaned and exported using the ‘multiExport' script [49]. Resulting image stacks were imported into VG Studio 2022.2 (Volume Graphics, Heidelberg, Germany) to create volume renders (figures 1–3; electronic supplementary material, S1).

**Figure 4.**
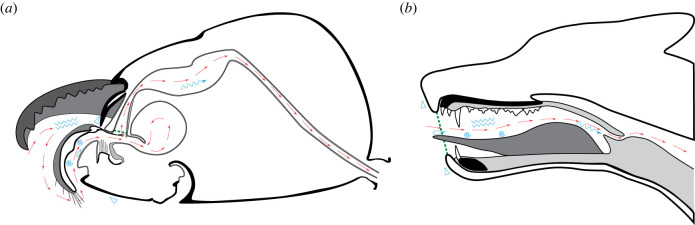
Schematized drawings of an ant (*a*) and a dog (*b*) head, illustrating the path of food through the feeding apparatus (red arrows). In the ant, most food presumably passes the infrabuccal pouch first before ingestion, but liquids may also be unhindered by the filter apparatus and pass straight through the mouth opening. Location of the functional mouth opening marked by green dashed line in both. Cyan symbols represent different functions and their locations. Zigzag lines: mechanical food processing/chewing. Triangles: dorsal and ventral closure of the preoral/oral space. Drop: licking of liquids. Spiral: food manipulation in the preoral/oral space. Wavy arrow: ingestion into the digestive tract, sucking pump in ants, tongue and pharyngeal muscles in dog/mammals.

Additionally, we used scanning electron microscopy images previously published by us [21] and Keller [40] to visualize details of the mouthparts (figure 3; electronic supplementary material, S2) and made drawings in Adobe Illustrator 2023 CC (Adobe Systems, San Jose, CA, USA) comparing food uptake in an ant and a dog (figure 4). Image plates were assembled in Adobe Photoshop CC 2023 and labels added in Adobe Illustrator 2023 CC. Specimen numbers of images by Keller are indicated in the respecitve figure legends and can be accessed through the advanced search on antweb.org.

Finally, we assembled interactive 3D surface models of the structures in Blender 3.4.1. (Blender Foundation, Amsterdam, The Netherlands) and uploaded them to Sketchfab. Links to the models can be found in the legend of figure 2.

## 3. Results and discussion

### (a) The feeding process in ants

Ant food uptake may be divided into two general modes. Solid food is first processed by the mandibles before transport into the preoral cavity and subsequent ingestion, while liquids are directly licked or sucked by the maxillolabial complex in combination with the sucking pump [29,30] (figure 1*a*). Liquid uptake has been extensively studied in the laboratory using sugar water. In nature, typical fluid sources are haemolymph of cut-up prey [50], plant juices when cutting leaves [51], extrafloral nectaries [24], trophobiont excretions [11] or regurgitated food from other adult or larval ants, or even larval haemolymph [52]. Ants may lick up liquids by rhythmically extending and retracting the tongue-like glossa, potentially supported by forewards and backwards movements of the whole maxillolabial complex. Alternatively, they may suck fluid more passively by inserting their maxillolabial complex into the liquid and holding it in place [29]. In both cases, liquid is initially taken up through adhesion generated by the hairy surface of the glossa. Other hairy structures on the mouthparts may also play a role [30,53,54]. When the glossa is licking, the galeae swipe across it as it is retracted, presumably helping to move the liquid further up into the preoral cavity [30]. From the preoral cavity, liquid is then sucked up through the mouth opening by the cephalic sucking pump [26,30].

Interestingly, the uptake mechanism that is used is both context- and species-dependent. Ponerine ant species generally perform licking movements when foraging for collecting food, and the liquid is then often collected between the mandibles [29]. Passive sucking, however, has been observed in the ponerine *Odontomachus chelifer* [28]. By contrast, formicine ants almost exclusively employ the sucking mode and only switch to licking when a drop of liquid is too small to insert the maxillolabial complex [29]. The rate of food uptake is variable in different ant species, but only a fraction of ant diversity has been studied so far [24,29]. Additionally, intraspecific variation in sucking pump activity rate and crop filling level can be related to factors like sugar concentration, viscosity and individual or colony starvation level [25–27,54,55].

Surprisingly, we did not find any thorough description of mouthpart movements for solid food uptake in the literature. Nevertheless, it appears clear that the first step for most ant species is to handle and process food items with their mandibles [5]. Many species use the toothed mandibular margin to grip and hold arthropod prey before cutting and chewing them into small pieces [56,57]. Harvester ants may even process solid seeds using the mandibles. They open them [12,58] and chew them into ‘ant bread' while adding saliva from the labium [59]. During handling with the mandibles, food is often touched with the palps of maxilla and labium [60], finger-like appendages with many sensory hairs [21]. This presumably allows ants to identify chemical and mechanical properties of their potential food.

Can adult ants directly ingest the processed solid food? Many observations report that not just seeds, but also solid parts of prey items are only pre-processed by the worker ants [58,61], and then given to their larvae for actual ingestion [62]. Adult ants have an effective filter apparatus consisting of rows of cuticular hairs surrounding their mouth entrance (figure 1*a*, insert). This filter prevents particles of different sizes (*ca* 150 µm in a formicine ant, down to *ca* 1 µm in a myrmicine ant) from entering the digestive tract [63,64]. However, there are contradicting observations of worker ants directly consuming their prey in the field [56]. Harvester ants ingest chunks of processed seeds and these were also found in their crop (social stomach) [12,59]. Other solid food particles were also found in the crop of more generalized species (e.g. *Technomyrmex* sp. [24]). This shows that some level of solid food uptake is evidently possible in adult ants, but how it can be ingested despite the mouth filter is poorly understood. Elucidating the role of ingestion in the processing of solid food by ants clearly requires further focused research attention.

As ants are eusocial insects, the social dimension also plays a crucial role in their feeding biology. As larvae often ingest solid food particles that may not be eaten by the adults [62,63] and some of this processed and metabolized food may then be shared back to the adults [52], larvae have been referred to as a ‘digestive caste' [6]. Food exchange also occurs among adults to different degrees in different lineages [52]. In oral food exchange, the glossae are joined together while the maxillolabial complex is held steady, indicating the sucking pump as the most important part of the feeding apparatus in this behaviour.

### (b) Morphology of the feeding system in ants

#### (i) The mandibles, a multipurpose tool

The mandibles of ants are the most visible and most well-studied tools among their mouthparts, forming gripping and sometimes grinding or cutting jaws. Two contributions [38,39] in this special issue focus specifically on the biomechanics of the mandibles, so only a very short overview of these structures is given here.

The mandibles are set on both sides of the oral foramen and their bases thus form the lateral closure of this space, flanking the other mouthparts. Ant mandibles have a relatively narrow basal stem compared with the usually broadened, triangular, concave blade. Owing to this shape, the mandibles always project distinctly in front of and above the other mouthparts (figure 1*a,b*). This means they can work relatively isolated from the other mouthparts, facilitating functions such as fighting, prey capture, food processing/chewing, nest building, carrying objects and nest-mates including brood items, and even communication [5]. During many of these tasks, the remaining mouthparts are tightly retracted into the oral foramen, keeping them out of the way and protecting them (figure 2*c*; electronic supplementary material, S1A).

Related to their functional diversity, ant mandibles are incredibly diverse in shape and size, ranging from broad grindstones to long, narrow pincers and spiked forks [17,33]. They are typically dicondylic, with movements constrained in a horizontal plane (but see [38]). A large closer and a smaller opener muscle move the mandible. The closer muscle consists of several different fibre types, indicating fine control of movements. Variation of fibre composition points to adaptation of this system to different ecologies in combination with the various mandible shapes [33–35].

#### (ii) The maxillolabial complex, the composite tongue of ants

As is typical for many insects, ants have two movable tools for food uptake, the paired maxillae (lower jaws) flanking the unpaired labium (tongue or lower lip). Both are more complicated than the mandibles as they consist of several movable parts, hinting at their evolutionary origin from segmented extremities [13]. While in most insects these two mouthparts move rather independently from each other, they form a closely connected and functionally integrated unit, the maxillolabial complex, in ants and other Hymenoptera. This connection is realized at two main points: a narrow membrane connects the bases of both parts, and extensions of thicker cuticle fuse on their back sides at around mid-length (figure 2*c*). In ants, this whole complex is also closely integrated with the labrum, as this ‘upper lip’ closes on top of the complex when it is retracted (figures 1*c* and 2*b*).

Both mouthparts consist of a small basal piece (cardo of maxilla and postmentum of labium) and a larger central plate (stipes of maxilla and prementum of labium) (figures 1 and 2), which are movable against each other. The only articulation of the whole complex with the head capsule are the bases of the club-shaped cardines (figure 2*d*; electronic supplementary material, S1B). The spatial configuration between cardo and stipes can be imagined as a V-shape (figure 2*a,d*), which flattens when the mouthparts are extended. The labial postmentum is only connected to the head through soft cuticle (figure 2*e*). Owing to this configuration, the whole complex can be extended as a unit, but there is likely only little independent movement of labium and maxillae (figure 1).

At their tips, the central plates carry the multisegmented, movable, antenna-like palps. Alongside the inner side of the palp are two softer, movable lobes. For the maxilla, these are galea and lacinia, which are individually movable parts in many insects. In ants, they instead form one connected and rather soft plate that bends over the labium, forming a narrow channel in-between (figures 1 and 2). The lobes at the tip of the labium are the paired glossae and paraglossae. In ants, there is only one glossa (figure 3*g*), although this is not the case in all Hymenoptera. Paraglossae may be present at its sides as tiny folds but are often missing completely. Directly behind the glossa is the opening of the salivary duct, which is flanked on both sides by brushes of thick hairs (figure 3*f*). Behind this opening, the labium is covered by a soft, tongue-like structure, the hypopharynx (figures 1*a* and 3*f*).

The maxillolabial complex is moved by a complex set of muscles (figure 2*d–f*). Only Paul *et al*. previously attempted to infer potential movements of the complex based on its musculature [31]. We will base our description on their interpretation with some updates based on our 3D models. The maxillolabial complex is opened by extensor muscles inserting at the base of the central pieces of both maxillae and labium, which rotate these pieces outward by pulling their bases upwards. Retractor muscles insert on the middle of the stipes and on the tip of the hypopharynx. An additional muscle inserts on the base of the maxillary cardo, which may rotate the maxilla outwards, but could also be involved in extension of the whole complex.

Inside the maxilla (figure 2*d*), two muscles (the second was discovered here for the first time and is not mentioned in previous descriptions) move the palp and insert at its base. One muscle inserts on the base of the galea. The galea is bent downwards when the mouthparts are retracted (figure 3*d*; electronic supplementary material, S1A), indicating that this muscle either keeps the galea pulled down or, more likely, pulls it upwards when the mouthparts are in use. A transverse muscle inserts on the base of the lacinia and likely pulls it downwards. Inside the labium, large glossa muscles insert at the back of the glossa and smaller paraglossa muscles insert at its front (figure 2*e*). They retract the glossa, which then protracts elastically.

This function is crucial in licking up liquid food as discussed above. While we did not find documentation of feeding motions for solid food, a potential pattern emerges based on the inventory of muscles. It appears likely that a combination of up and down movements of the galeolacinial lobe and forewards and backwards movements of the whole complex are the main possible movements suited to transport food items. A pair of salivarium muscles inside the hypopharynx insert on the salivary duct, extending its opening to allow gland secretion release. Only one muscle moves the labial palp. The internal muscles in the first few segments of both labial and maxillary palp allow them independent movement.

The equipment of cuticular hairs on the different parts of the complex is functionally highly relevant and variable across different ants. An overview of some of the most important hair groups is given in figure 3; their variability is indicated in electronic supplementary material, figure S2. A comb of blunt hairs is uniformly present on the inner side of the galea (figure 3*a*). This comb may play a role especially in grooming behaviour when other body parts are pulled along the galeae [5,16]. The tip of the galea is covered in a brush of hairs with various densities, shapes and sizes (figure 3*d*, electronic supplementary material, S2D,E). As the galea is the most likely structure to be able to shovel solid food, these hairs may influence how such a function is performed. The lacinial margin has a comb of hairs of varying size, shape and density (figure 3*c*; electronic supplementary material, S2). When the mouthparts are extended, this comb rests in front of the mouth opening (figure 1*a*), indicating that it plays a role in the preoral filter mechanism. Since especially thick spines form this comb in many predacious ant species (figure 3*c*), its structure may be related to feeding ecology.

The glossa surface is covered in transverse rows of cuticular projections (microtrichia) to increase its surface area (figure 3*g*). These projections also vary in shape, size and density, with potential implications for feeding ecology [30]. Finally, the palps of the maxilla and labium are variable not only in their size and segment number, but also in the density and types of sensory hairs on their surface (figure 3*h*; electronic supplementary material, S2), which implies differences in how the palps are used for chemical and mechanical sensing and in communication [5]. Fully deciphering the functional role of the different hair groups and their variations will likely be challenging but would improve our understanding of mouthpart function and adaptation.

#### (iii) The labrum, a protective plate

The labrum is a cuticular plate of varying shape and size. It broadly attaches to the upper margin of the oral foramen between the mandibles through a thin, flexible band of cuticle, allowing forewards and backwards movements (figure 1*c*). It usually hangs straight downward in ants and thus covers the upper parts of the maxillolabial complex when retracted. A pair of muscles attach basally at its sides. This labral retractor (electronic supplementary material, figure S1C) likely pulls the labrum downward [31]. Labral opening may be passive through pushing of the maxillolabial complex as it extends.

The labrum is typically rectangular to trapezoidal, usually with a bilobed distal margin [17]. The enlarged labrum of, e.g. army ants, implies improved protection of the mouthparts [40]. The elongated, pointed labrum of ‘dirt ants' (Basicerotini) was hypothesized to be involved in prey capture [65]. In some trap jaw ants such as the genera *Strumigenys* and *Daceton*, the labrum is modified to serve as a latch mechanism for the mandibles, to enable their power-amplified prey capture strike [32]. Rows of stout setae (chetae) occur on the external labral surface in some predatory ants (e.g. Amblyoponinae, Leptanillinae, stem ants), and may improve grip onto prey and other objects [19,40].

#### (iv) The preoral cavity, the filtration chamber

The mouthparts surround an open space in front of the functional mouth opening, which is known as the preoral cavity (figure 2*f*). Food passes through this space, is filtered, and may be temporarily stored here. It is completely sealed off when the mouthparts are closed (figure 2*b*; electronic supplementary material, S1A). The cavity's upper wall originates on the inner labral margin and is called the epipharynx. The lower wall is the hypopharynx. The front part of the hypopharynx forms the tongue-like structure above the labium, but behind this it extends into a large sac, the infrabuccal pouch (figure 1*a*). Most of the preoral space consists of thin, flexible cuticle. Right above the infrabuccal pouch, epi- and hypopharynx meet to form the broad, slit-shaped mouth, which opens into a narrow buccal tube (figure 1*a*, insert).

Most of the preoral cavity is covered in tiny cuticular hairs. This cover has been documented in detail for only a few ant species [41,43,44], but the general design appears rather conserved. The epipharynx is sparsely covered in tiny hairs, often arranged in comb-like rows, while the hypopharyngeal tongue is densely covered in hairs, varying from long to short from front to back (figure 3*f*). The inside of the infrabuccal pouch is mostly smooth. Dense brushes of long hairs are distributed on the dorsal and ventral wall of the mouth opening (figure 3*b*), forming almost a curtain in front of the slit. The inside of the buccal tube is set with rows of microtrichial combs of various length [20–22,41,44].

This hair-cover makes the preoral cavity into an effective filtration device. The infrabuccal pocket serves as a collecting reservoir for filtered particles, from either food or dirt from the environment, which may also come from self or nest-mate grooming [44]. The particles are formed into a pellet which is periodically ejected, about once every 24 h [41]. A multitude of bacteria were found inside the pellet, indicating potential extraoral digestion [42], although the time pellets spend in the pouch may be too short for this to occur [41]. However, several studies have found digestive enzymes in the salivary secretion of ants [61,66], so some level of digestion within the infrabuccal pocket appears likely. Some ants feed the pellets to their larvae [62,63].

As the preoral space is not operated directly by any muscles, it only deforms passively when the other parts are moved. This raises the question of how the pellet accumulating in the infrabuccal pouch is ejected. Closer observations of mouthpart movements may help in resolving this question.

### (c) The sucking pump, ‘where ingestion happens'

Following the short and narrow buccal tube that reaches into the preoral space, the alimentary canal widens into the cephalic sucking pump (figure 1*a*). This structure creates suction through volume expansion, ingesting liquid or small particles from the preoral cavity or directly sucking liquid from external sources if the mouthparts are completely submerged. Most of the pump is made of thin, flexible cuticle, but its sides are stabilized by thick cuticular bars called the ‘oral arms' (electronic supplementary material, figure S1C,D). The arms curve from the bottom front of the pump to its upper back. The arms' posterior ends form variously shaped plates and processes that serve as points of muscle attachment [19]. Previous functional interpretations of sucking pump muscles are even more limited than for the maxillolabial complex, given its inaccessibility inside of the head [31]. We will give a short, updated view on potential pump muscle functions here, but focused study will be needed to improve our understanding.

A series of five muscles insert on the upper side of the sucking pump. One of them attaches to the buccal tube (figure 2*f*) and likely opens the mouth. The remaining four dorsal muscles are dilators that expand the pump to create suction and take in food from the preoral space/buccal tube together with the single ventral dilator (figure 2*f*). Two further muscles that originate on the head capsule insert on the plates of the oral arms, one from the front and one from the back (oral arm m, electronic supplementary material, figure S1C). These muscles probably pull the whole pump forewards and backwards, respectively. As antagonists to the dilators, large longitudinal and transverse muscles on the dorsal side of the pump contract it.

This account is likely a simplification and the true dynamics of pump movements could be complicated by activation of different combinations of the many muscles as well as the patterns of soft and hard parts of the pump wall. The shape of the oral arms [18,20,21], the pump's general size and proportions [16,18,20–23], as well as the proportions of different muscles, [18,20–23,30] are known to be variable. Explanations for these differences may be found in some of the species-specific food uptake performance differences observed in ants, but could also be related to social behaviour. Trophallaxis, the sharing of fluids with nest-mates, requires regurgitation and may thus also be affected by pump architecture [52].

### (d) Comparison with other insects

General mouthpart structure is similar between ants and related hymenopterans such as vespid [67,68] or sphecid wasps [19,69], and many similarities exist even with more distantly related parasitoid wasps [70]. The most prominent differences of closer relatives such as sphecids are the larger paraglossae, the hair-cover of the maxillolabial complex, the proportions and attachment angles of some muscles (especially the labial extensor), and an overall more open condition of the complex, as it is not usually retracted completely behind the labrum [19]. As in ants, food is filtered in the preoral cavity, which was studied in some detail for vespids [71]. A phenomenon that occurs in many wasps and bees but is completely absent in ants is the elongation of the maxillolabial complex and formation of a specialized, tube-like, proboscis [72]. While different parts of the complex may be modified to form such sucking tubes, their function is usually related to specialized feeding on nectar from flowers, which is not an important food source for most ants [6]. In other insect groups such as Lepidoptera, Diptera and Hemiptera, even more derived structures for liquid feeding have evolved independently. Intricate piercing, sucking and lapping tools are constructed through variable fusion and reduction of some parts while others are strongly enlarged and/or modified [13]. The butterfly proboscis as an example is entirely composed of the maxillary galeae, with the mandibles reduced to small rudiments.

Ants may be seen as intermediate between such specialized liquid feeders and insects with more generalized biting–chewing mouthparts. A good example of the latter are cockroaches [73]. The American cockroach is so far the only insect for which a detailed kinematic analysis of mouthpart movements has been performed by employing cineradiography [74]. In contrast to ants, the cockroach labrum lies on top of the mandibles rather than behind them, and maxillae and labium are not fused, moving more independently. The mandibles work inside the preoral space in synchronized movements with maxillae and labium, in contrast to the more independent action in front of the other mouthparts in ants. The typical cockroach food uptake sequence, which can be extrapolated to most biting–chewing (or ‘orthopteroid') insects, starts with grabbing food with the mandibles. The mandibles are more or less strongly divided into a distal incisival part to grab, pierce and cut objects, and a molar part for grinding. Food is transported in between the molar parts by movements of the mandibles and maxillae, where it is chewed until further transport towards the mouth opening using maxillae, labium and hypopharynx. For their more active grabbing and shovelling, the cockroach maxillae have a harder, tooth-like lacinia than the soft one in ants. The hypopharynx is less fused to the labium and can be moved independently to transport food towards the mouth like a tongue. Correlated with these overall more complex movements, the mouthparts are operated by 36 muscles in cockroaches [73] (maxillae, labium, part of the hypopharynx) compared with the maximum of 20 operating the ant maxillolabial complex.

By using the mandibles in front of and more independent from the other mouthparts, adult ants and other hymenopterans likely achieve more specialized function, with the mandible as multipurpose tool and the maxillolabial complex for food uptake. Especially in a social context, this probably contributes to the ability to perform complex manipulations with the mandibles when constructing nests, catching prey, caring for brood and defending the colony.

### (e) Comparison with vertebrates

Given their evolutionary distance, it is unsurprising that ants and vertebrates have found very different solutions to the problem of processing food and transporting it into their digestive system. The basis for these different solutions is the exoskeleton of arthropods with many multisegmented extremities that can be modified for most important life functions, and the endoskeleton of vertebrates with a more limited number of extremities.

While extremities may be used for food uptake in some vertebrates like primates, this is more commonly achieved directly by the lips, tongue and teeth of the mouth [75]. The teeth are set in a bony jaw, which is the basic tool for vertebrates, or at least tetrapods, to process food inside the oral cavity [76] (figure 4*b*). While suction feeding through expansion of the jaw and pharyngeal apparatus is the major way of food uptake and transport in aquatic vertebrates [77], a muscular tongue is crucial for this in many terrestrial species [78].

Ants, like other arthropods, instead use the modified extremities in front of their mouth opening for all kinds of food handling and processing. Rather than inside the mouth, they thus process and handle their food mostly in a preoral space. The actual food uptake then functions through suction which is generated by dilation of the sucking pump (figure 4*a*).

Nevertheless, some analogies and similar principles can be found. Processing of food by the mandible is similar to biting and chewing in vertebrates. Licking with the glossa is similar to licking with the tongue, and shovelling of food with the maxillae is similar to food manipulation with the tongue. Just as cheeks, lips and jaws can close off the vertebrate oral space, the labrum and maxillolabial complex can close off the preoral space of ants.

Research on food processing and the evolution of feeding structures has progressed to very detailed questions in many vertebrates compared with ants and other insects. In this special issue alone, we find contributions on such intricate topics as 3D jaw kinematics of basal mammalians [79] and soft tissue dynamics during mammalian mastication [80].

### (f) Future directions

The basic principles of ant food uptake are well understood. Furthermore, mouthpart morphology has been described for many species, for some of them to a high degree of detail. Nevertheless, some gaping holes in our understanding on ant feeding are apparent. Most prominently, we do not know how the maxillolabial complex moves to transport solid food particles in the preoral space. Basic kinematic research would be highly useful to clarify this problem. While ideas exist for the functions of the various groups of hairs on the maxillolabial complex, hardly any of these have been observed or tested or analysed in relation to differences in feeding ecology or social behaviour.

Other fundamental questions remain. How do ants take up solid food when even particles of minute size are filtered by the preoral space? How is the complicated musculature of the sucking pump coordinated? What is the role of the sclerotized oral arms in pump function? Are differences in pump muscles and sclerites related to feeding performance, food preferences and/or social behaviour such as trophallaxis? How important is the ability of ants to share food with their larvae and thus divide different roles in food processing to different colony members for ant eusociality?

Comprehensive investigation of the morphology and physiology of the ant feeding apparatus would provide more fundamental understanding of the diversification of this ecologically dominant clade. Moreover, understanding the case of ants may also give us new insights into the general principles of food uptake, one of the most fundamental life functions for heterotrophic organisms, and how different lineages cope with the challenges of this task.

## Data Availability

Data used for the 3D models in this article are published from the Zenodo repository: https://zenodo.org/record/4623822 [81] and from: https://zenodo.org/record/3786977 [82].
